# Prevalence of self-reported hypertension and its relation to dietary habits, in adults; a nutrition & health survey in Greece

**DOI:** 10.1186/1471-2458-6-206

**Published:** 2006-08-13

**Authors:** Christos Pitsavos, George A Milias, Demosthenes B Panagiotakos, Dimitra Xenaki, George Panagopoulos, Christodoulos Stefanadis

**Affiliations:** 1 First Cardiology Clinic, School of Medicine, University of Athens, Greece; 2 Department of Nutrition – Dietetics, Harokopio University, Athens, Greece; 3 Unilever Institute, Athens, Greece

## Abstract

**Background:**

Hypertension leads to many degenerative diseases, the most common being cardiovascular in origin. This study has been designed to estimate the prevalence of self-reported hypertension in a random nationwide sample of adult Greek population, while focus was set to the assessment of participants' nutritional habits in relation to their hypertension status.

**Methods:**

A random-digit dialed telephone survey. Based on a multistage, stratified sampling, 5003 adults (18 – 74 yr) participated (men: 48.8%, women: 51.2%). All participants were interviewed via telephone by trained personnel who used a standard questionnaire. The questionnaire included demographic and socioeconomic characteristics, medical history, lifestyle habits and nutritional assessment.

**Results:**

The prevalence of self-reported hypertension was 13.3% in men and 17.7% in women (P < 0.001). Furthermore, women reported higher values of systolic blood pressure (180 ± 27 mmHg) than men (169 ± 24 mmHg). Positive relationships were found between hypertension status and the prevalence of the rest investigated health conditions (i.e. hypercholesterolaemia, diabetes mellitus, renal failure and obesity). Nutritional assessment showed that consumption of fish, fruits and juices, cereals, and low fat milk and yogurt was significantly higher among hypertensive subjects while the opposite was observed for food items as red meat, pork, egg, pasta and rice, full fat dairy products and desserts.

**Conclusion:**

Hypertension seems to be a serious public health problem in Greece. It is encouraging that hypertensives may have started adopting some more healthy nutritional behaviour compared to normotensive ones. However, they can gain significant benefits regarding to blood pressure control, if they increase the level of compliance with dietary recommendations.

## Background

High blood pressure [1] has been identified as a major risk factor for stroke, congestive heart failure, renal disease and myocardial infarction [2,3]. According to World Health Organization the number of people with high blood pressure levels, worldwide, is estimated to be about 600 million and the annual mortality attributable to hypertension is estimated to be about 7.14 million deaths. As far as the European developed countries is concerned, it is estimated that hypertension is responsible for about 17% of total annual mortality or in other words, for approximately 680 thousands deaths annually [4]. However, the prevalence of hypertension shows a significant variability among different countries. For instance, according to the work of Wolf-Maier and colleagues [5] prevalence of hypertension was found to be 37.7% in Italy, 38.4% in Sweden, 41.7% in England, 48.7% in Finland, 46.8% in Spain, and 55.3% in Germany. In the same review paper, it is stated that the mean prevalence of hypertension is higher in Europe (approximately 44%) compared to the US (approximately 28%). Despite the motivational finding, that treating hypertension is associated with about a 40% reduction in the risk of stroke and about a 15% reduction in the risk of myocardial infarction [6], only approximately 12.5% of hypertensive subjects show adequate control of their blood pressure [7-9]. The risk of cardiovascular complications and organ damage in persons with high blood pressure is increased when other risk factors such as smoking, obesity, inappropriate dietary habits and physical inactivity are also present. In the opposite, the benefits from healthy dietary patterns on blood pressure control have been reported in several studies [10-15]. Therefore, appropriate nutrition related life-style modifications should be employed at all stages of high blood pressure managing. This is in accordance with the National High Blood Pressure Education Program [16] in US which emphasizes 6 approaches with proven efficacy for prevention of hypertension: engage in moderate physical activity; maintain normal body weight; limit alcohol consumption; reduce sodium intake; maintain adequate intake of potassium; and consume a diet rich in fruits, vegetables, and low-fat dairy products and reduced in saturated and total fat.

Current data regarding the epidemiology of hypertension in the Greek population are lacking. Therefore the primary aim of this study was to evaluate the prevalence of hypertension in a representative nationwide sample of Greek adults and to investigate the nutritional habits of the participants in relation to their hypertension status.

## Methods

### Study sample

During autumn of 2004, 62,538 men and women from all Greek regions were randomly selected through telephone catalogues in order to participate in a random-digit dialed (RDD) telephone survey. From the design of the study it has been decided that the sample should be stratified by age group, sex and Greek region in order to be more representative. The randomized procedure was based on the following rule: we selected 1 every 10 names of each particular area using a sequence of random numbers. Furthermore, only one person per household was decided to be interviewed, by trained personnel who used a standard questionnaire. Of the contacted people, 48% did not answer the telephone-call because of various reasons (i.e. 39.1% absences, 4.5% occupied phones, 3.9% wrong numbers recorded in the catalogues); 14.3% of the contacted persons were not suitable for interviewing (i.e. children or house girls etc); 11% were not suitable based on the quotas (i.e. they did not correspond to the sampling targets we had pre-designed for each area, in order the stratified sample to be as much representative as we can) and 0.4% were calls were interrupted due to unknown reasons. Thus, 16,760 of the contacted adult people were eligible for the study. Of them, 5003 (18 – 74 years, men: 48.8%) agreed to participate (participation rate: 30%); the participation rate is considered acceptable since according to the Council of American Survey Research Organizations (CASRO) the response rate estimation of a RDD telephone surveys [17], based on our data, should be 29.59%. All participants were informed by the interviewer about the aims and procedures of the study. However, since this was a telephone interview no written informed consent was obtained from the participants. The ethical committee of our Institution approved the design of the study.

### Investigated measurements

The questionnaire addressed individuals' demographic and socioeconomic characteristics (age, sex, years of education, type of occupation and residence), information relevant to health status with focus on arterial hypertension (defined as: systolic/diastolic blood pressures > 140/90 mmHg [1] or use of anti-hypertensive treatment), hypercholesterolemia (defined as: total serum cholesterol levels > 200 mg/dl [18] or use of lipid lowering agents) and diabetes mellitus (defined as: fasting blood glucose > 125 mg/dl [19] or use of hypoglycemic treatment), food consumption pattern, other lifestyle habits, such as smoking habits, level of physical activity as well as characteristics related to body composition (recalled weight and height). Family history of all the aforementioned health problems was also recorded (type of answer: yes/no/don't know for father, mother, brother and sister).

Specifically for the assessment of hypertension status, the subjects were considered as hypertensive if they were reporting that they had been previously diagnosed by a registered physician (type of answer: yes/no). In case of positive answer, they were asked to report an average value of their systolic blood pressure during hypertensive episodes and to provide data relevant to the selected way of treatment (antihypertensive medication or/and diet or no treatment, years of treatment).

Relatively to the nutritional component of the questionnaire, the participants were asked to report their average weekly consumption (in servings) of a list of food items of all different food groups. The educational level of the participants was measured by the years of schooling (Group I: < 9 years, Group II: up to high school or technical colleges (10 – 14 years) and Group III: university). Current smokers were defined as those who smoked at least one cigarette per day; never smokers those who have never tried a cigarette in their life and former smokers were defined as those who had stopped smoking in the past. Physically active were considered all the participants who reported either that they participated in an exercise program (such as gym, sports, jogging etc.) during their leisure time at least once a week, or that their occupation presupposes certain level of physical fatigue. The rest of the subjects were defined as physically inactive. Finally, we used data of self reported weight and height to calculate body mass index (BMI). Participants were classified as obese if BMI was ≥ 30 kg/m^2^.

### Statistical analysis

Continuous variables are presented as mean values ± standard deviation, while qualitative variables are presented as absolute and relative frequencies. Relationships between categorical variables were tested by the use of contingency tables and the calculation of Pearson's chi-squared test. All comparisons among paired groups of sample (males – females, normotensives – hypertensives) in relation to normally distributed continuous variables were performed using the student t-test. Every reported P-value is based on two-sided tests and compared to a significance level of 1%. SPSS version 13 (Statistical Package for Social Sciences, SPSS Inc, Chicago, Illinois, U.S.A.) software was used for all the statistical calculations.

## Results

The proportional distribution of the present study's participants and of the Greek population (provided by the National Statistical Service according to the census of 2001), by sex and age, is presented in Table 1. No differences were observed regarding age-sex between the sampled and the population distribution (chi-square for men = 0.006, p > 0.99 and chi-square for women = 0.005, p > 0.99), indicating that the stratified by region sample was representative of the total population in terms of age and sex.

The characteristics (demographic characteristics, lifestyle habits and information from the medical history) of the study's participants in relation to sex are presented in Table 2. The mean age of men was slightly smaller than that of the women (43.7 ± 16.1 and 44.7 ± 16.0 years, respectively). Additionally, men were more educated and physically active, as well as more frequently current and former smokers.

Analysis of data relevant to the medical history showed statistically significant difference among men and women in relation to hypertension status and prevalence of hypercholesterolaemia, but not to prevalence of obesity, diabetes mellitus and renal failure. In particular, 325 men (13.3%) and 455 women (17.7%) reported that they were hypertensive, while 400 men (16.4%) and 560 women (21.8%) reported that they had hypercholesterolaemia. Strong positive relations were found between hypertension status and the prevalence of the rest investigated health conditions (i.e. hypercholesterolaemia, diabetes mellitus, renal failure and obesity) in the overall sample. The prevalence's values of these health conditions among normotensive and hypertensive subjects are presented in Table 3 (all *P*s < 0.001).

The distribution of hypertensive study's participants by age group is presented in Table 4. Ío significant difference was found among men and women in the first five age groups (total range 18 – 64yr) while in the older age group examined (65 – 74yr), hypertension was significantly more prevalent in women comparing to men.

Statistically significant difference (P < 0.001) was observed among women and men participants in relation to the reported values of blood pressure, when they faced episodes of hypertension. In particular, women had higher values of systolic blood pressure (180 ± 27 mmHg) than men (169 ± 24 mmHg). However, no difference was found relatively to parameters as "number of years that they have the problem" (men: 8.6 ± 7.8 yr, women: 9.4 ± 8.2 yr, P = 0.155) and "number of years that they use antihypertensive medication" (men: 7.5 ± 6.7 yr, women: 8.4 ± 7.6 yr, P = 0.126). Furthermore, analysis of participants' family history of hypertension showed a positive association (P < 0.001) only between brothers (independently of sex) and not between parents and descendants. Specifically, only 1.32% of normotensive participants have a hypertensive brother or a sister, while 6.56% of hypertensive participants have a brother or a sister who faces the same problem.

Several associations were also detected between nutritional habits and hypertension status. The average weekly consumption (expressed in servings) of the investigated food groups by normotensive and hypertensive subjects is presented in Table 5. With the exception of some food groups or items i.e. chicken (P = 0.298), vegetables (P = 0.272), white cheese (P = 0.055) and legumes (P = 0.063), hypertensive participants differ significantly when compared to normotensive ones in relation to consumption of the food groups tested. In details, data came out after the analysis of the semi-quantitative food frequency questionnaires showed that hypertensives consume more frequently items from the groups of fish (P < 0.001), bread and cereals (P = 0.011), fruits and juices (P < 0.001) and low fat milk and yogurt (P < 0.001), while normotensive participants have greater consumption of red meat (P < 0.001), pork (P < 0.001), egg (P < 0.001), pasta and rice (P < 0.001), potatoes (P < 0.001), full fat milk and yogurt (P < 0.001), yellow cheese (P <0.001) and desserts or ice creams (P < 0.001).

## Discussion

This study provided data on the prevalence of self – reported hypertension among a nationwide sample of about 5000 Greek adults. Furthermore the nutritional habits of the participants were evaluated in relation to their blood pressure status. The prevalence of self-reported hypertension was about 13% in men and 18% in women, denoting a serious public health problem in Greece. However, it is encouraging that hypertensive subjects may have started adopting some more healthy nutritional behaviour compared to normotensive ones.

Only a few epidemiological studies [20-23] have provided data regarding the prevalence of hypertension in Greek population. Two of them, the ATTICA study [20] and the Greek EPIC study [21], are well organized and large – scale health surveys and provided information about the prevalence and awareness of high blood pressure levels in representative Greek samples. In particular, the ATTICA study is a health and nutrition survey that enrolled 3042 adult men and women, without clinical evidence of cardiovascular disease, from the province of Attica in which Athens, the capital of the country, is located. The sampling was random and multistage, and was based on the age-sex distribution of the province of Attica, provided by the National Statistical Service according to the census of 2001. Additionally, the EPIC study is a multi-country, prospective cohort study that was conducted in 22 research centres in 10 European countries, examining the role of dietary, lifestyle, and environmental factors in the aetiology of cancer and other chronic diseases. In Greece, the EPIC study started in 1994 and is being conducted by the Department of Hygiene and Epidemiology of the University of Athens. The subjects of the EPIC study were volunteers (n = 26,913), aged 20–86 years and recruited from several regions of Greece. Arterial blood pressure measurements were taken in both of the aforementioned studies in order to diagnose hypertension among participants.

According to the ATTICA study, the prevalence of hypertension was 37.5% for men and 25% for women, while according to the Greek EPIC study, the respective figures were 40.2% and 38.9%. Another local, small – scale, observational study (Didima study) [22], showed similar to the ATTICA study results when evaluated the prevalence of hypertension (30% in men and 27% in women). The present study estimated that the prevalence of self-reported hypertension was much lower comparing to data provided from the studies mentioned above (13.3% for men and 17.7% for women). This large difference among these specific studies can be possibly ascribed to the selected way of identifying hypertensive participants in the present study. Due to the fact that the participants self – reported whether they are hypertensives (without clinical examination), it is actually likely that the prevalence's figures were underestimated as it is generally accepted that there is a large group of hypertensive subjects unaware of their condition (according to the ATTICA study: 68% in men and 54% in women [24]). However, studying population-based surveys the calculated prevalence may be an overestimate, as blood pressure measurement was performed once during the study and numerous reasons for elevated blood pressure readings may have been present, including the white coat phenomenon [25].

It is also noteworthy the finding that hypertension is more prevalent in women than in men, something that is in contrast with the results from the other similar surveys (ATTICA, EPIC and Didima). However, in the EPIC study it is stated that although the prevalence of hypertension before the age of 55 years is higher among men than among women, it is slightly higher among women thereafter. A different explanation of the above finding could possibly be the different level of high blood pressure awareness between the two sexes, as it has been suggested that hypertensive women show higher level of awareness than men [26]. Another finding of this study, which is in accordance with the results from the aforementioned studies, is that the prevalence of the discussed condition increases with age (Table 4). Particularly, distribution of hypertensive study's participants by age showed that prevalence's figures among consecutive age groups (decades) differ approximately per 15 percentage units, with highest values observed in the older age group (65 – 74 yr) examined (men: 39.5%, women: 49.6%).

The significant strong relationship observed between prevalence of hypertension and hypercholesterolaemia, diabetes mellitus and obesity (Table 3) confirm the frequent conclusion from other studies [27-29], that hypertensives usually, apart from high blood pressure, have additional cardiovascular risk factors. Although elevated levels of blood pressure and cholesterol are known as two of the most important risk factors of coronary heart disease [29], the strong relationships detected between hypertension and diabetes mellitus should not be underestimated, because evidence of diabetes substantially increases cardiovascular disease risk in hypertensive [30] while the risk of vascular complications in diabetes is related to the level of blood pressure [31]. Furthermore, obesity which was found to be more prevalent in the present survey's subgroup of hypertensive participants, also enhances total cardiovascular risk possibly by increasing low density lipoproteins-cholesterol (LDL-C) levels, reducing high density lipoproteins – cholesterol (HDL-C) levels, diminishing glucose tolerance and predisposing to the development of left ventricular hypertrophy, according to previous studies [32,33]. Above findings in combination to one of the conclusions from ATTICA study, that only about one out of six Greek hypertensives is adequately controlled, emphasize in the need for policies in Greece for the detection and control of hypertension similar to those that US as well as some other countries have adopted for years [7,34,35].

Diet and nutrition have been extensively investigated as risk factors for major cardiovascular diseases like coronary heart disease and stroke and are also linked to other cardiovascular risk factors like diabetes, high blood pressure and obesity [7,8,10,11]. Furthermore, arterial pressure regulation has been linked to a variety of nutrients and nutritional issues. Current guidelines for the management of hypertension emphasize the importance of achieving several nutritional goals simultaneously. In particular, according to the recent "2004 Canadian recommendations for the management of hypertension"[36], apart from suggestions relevant to improvement of physical fitness and stress management, nutritional guidelines are highlighted. Specifically, key recommendations include the following: an ideal body weight (BMI: 18.5 kg/m^2 ^to 24.9 kg/m^2^) should be maintained and weight loss strategies should use a multidisciplinary approach; alcohol consumption should be limited to two drinks or fewer per day, and weekly intake should not exceed 14 standard drinks for men and 9 standard drinks for women; a reduced fat, low cholesterol diet that emphasizes fruits, vegetables and low fat dairy products, and maintains an adequate intake of potassium, magnesium and calcium, should be followed; salt intake should be restricted to 65 mmol/day to 100 mmol/day in hypertensive individuals and less than 100 mmol/day in normotensive individuals at high risk for developing hypertension. It is also stated that the above lifestyle modifications should be extended to non-hypertensive individuals who are at risk for developing high blood pressure.

In the present study, the expected higher prevalence of obesity in the subgroup of hypertensive participants was confirmed, as well as the greater values of BMI in the same subgroup (Table 3). This finding shows that although hypertensive participants are aware of their hypertensive status, they have not managed yet to approach an ideal body weight. However, data came out after the analysis of food frequency questionnaires reveal that they may have started adopting some more healthy nutritional behaviour compared to normotensive ones. As it was mentioned in the results section, consumption of fish, fruits and juices, cereals, and low fat milk and yogurt was found to be higher among hypertensive subjects while the opposite was observed for food items as red meat, pork, egg, pasta and rice, full fat dairy products and desserts (Table 5), something that is encouraging relatively to their attempts of controlling their blood pressure in a better way. However, the reported quantities consumed are quite different than recommended ones, according to the beneficial dietary pattern of Mediterranean Diet [14,15,37] which among others highlights the importance of adequate consumption of vegetables and legumes.

It is noteworthy that eggs and full fat dairy products are included in the list of food items preferred more by present study's subgroup of normotensives. A first possible explanation could be that hypertensive participants try to follow more strictly the recommendations of nutritional experts. However, composition of these specific food items may reveal a better explanation of the observed relationships. In particular, peptides formed during the digestion of milk [38-41] and egg proteins [42] and oligopeptides from chicken egg yolk [43,44], have been demonstrated to have a blood pressure lowering effect in human, possibly via their strong angiotensin I – converting enzyme (ACE) inhibitory activity. So, it is possible this dietary habit observed among normotensive subjects to be able of providing a prophylactic effect against hypertension. Taking under consideration recent studies which demonstrated that there are no convincing evidence of an increased risk of vascular disease from milk [45,46] and egg [47] consumption, future focused research is needed before a possible update of global recommendations in relation to optimum consumption of egg and dairy products takes place.

## Limitations

This study as a cross-sectional one cannot establish causal relations, but only generate hypothesis that could be evaluated by future prospective randomized trials. Additionally, the applied method of self – reporting hypertension status neither is able to provide data in relation to level of hypertension's awareness (thus the evaluated prevalence of hypertension may be underestimated), nor can be as accurate as clinical examination. However, validity studies suggest that self – reported hypertension may be used for surveillance of hypertension trends in the absence of measured blood pressure [48]. For example, according to a recent survey [49], the results of which were based on blood pressure measurements, it was estimated that approximately 27 % of the US adults had hypertension and that among them about 69 % were aware of their status (corresponding to about 19 % of the study sample). In another study (with no blood pressure measurements) Ayala and colleagues [50] reported a prevalence of 20 % for self-reported hypertension among US adults, which is close with the expected value of 19%. Meanwhile, caution has to be given when interpreting results derived from self-reporting quantifiable variables, due to low reliability and validity of the self-reporting method. For instance, self-reported BMI tends to be underestimated in those with higher values. Thus, the rate of obesity is most probably underestimated [51,52]. Another methodological limitation of the present study is that semi-quantitative food frequency questionnaires in general cannot be used for subsequent analysis of specific nutrients, so conclusions can be expressed only in relation to food groups or items tested. Finally, the participation rate, although small (i.e. 30%) is considered acceptable in RDD telephone surveys. Unfortunately, no sensitivity analysis could be performed since no information was recorded by the people that they did not want to participate. Moreover, the statistical analysis showed that the selected sample shares the same distribution of age-group and sex as the total population.

## Conclusion

According to presented data, hypertension can be considered as a major public health problem in Greece, albeit the estimated prevalence is relatively low when compared with the rest European developed countries. Possibly the adoption of "western" dietary patterns by Greeks during last decades, had a strong causative role for this phenomenon. However, this problem can be prevented or controlled by complementary to clinical management application of strategies that target the general population. Among non – pharmacological approaches, nutritional interventions, that will incorporate the newest relevant scientific knowledge, are extremely challenging in the primary and secondary prevention of hypertension. Moreover a finding of this work, which implies certain actions towards health policy and quality improvement, is that although hypertensive participants seem to be aware of their condition, they have not managed as yet to approach an ideal body weight. All these carry an important message for primary care physicians and other health care scientists and reveals the value of the spread and understanding of European guidelines on cardiovascular disease prevention [53]. This study opens also room for further research with the same methodology that will explore whether patients with hypertension were seeking proper care by their personal physician and discuss to what extent they complied with their physicians' recommendations.

## Competing interests

The author(s) declare that they have no competing interests.

## Authors' contributions

CP: had the conception of the study, drafted the manuscript and given the final approval of the version to be published, GM: has been involved in concept of the study, performed the data analysis, and drafted the manuscript, DP: had the conception and design of the study, performed the data analysis and interpretation of the results, drafted the manuscript and given the final approval of the version to be published, DX, GP, CS: have made substantial contributions to conception and design of the study, have been involved in revising the manuscript critically for important intellectual content; and have given final approval of the version to be published.

## Pre-publication history

The pre-publication history for this paper can be accessed here:



## Figures and Tables

**Table 1:** Proportional distribution (in percentages) of the Greek population, aged ≥ 18 years and of the present study's participants (N = 5003), by sex and age

	**Study's Population**		Total Greek Population *	
**Age (years)**	Men	Women	Men	Women
18–24	11.2 %	10.0 %	13.7 %	12.4 %
25–34	22.5 %	21.0 %	21.2 %	19.9 %
35–44	20.0 %	19.7 %	19.4 %	19.3 %
45–54	17.8 %	17.7 %	16.9 %	16.9 %
55–64	14.6 %	15.8 %	14.7 %	15.8 %
65–74	13.8 %	15.8 %	14.1 %	15.7 %

* Data source: National Statistical Service, census of 2001

**Table 2:** Characteristics of the study's participants (% by sex)

	Men (n = 2439)	Women (n = 2564)	P
Age (years)	43.7 ± 16.1	44.7 ± 16.0	0.025
Education status ^a^			< 0.001
*Group I*	710 (29.1 %)	997 (38.9 %)	
*Group II*	763 (31.3 %)	787 (30.7 %)	
*Group III*	966 (39.6 %)	779 (30.4 %)	
Physical status			< 0.001
*Sedentary*	1664 (67.8 %)	2110 (82.3 %)	
*Physically active*	784 (32.2 %)	454 (17.7 %)	
Smoking habits			< 0.001
Never smoker	700 (28.7 %)	1381 (53.9 %)	
*Current smoker*	1176 (48.2 %)	901 (35.1 %)	
*Former smoker*	563 (23.1 %)	282 (11.0 %)	
BMI (Kg/m^2^) ^b^	26.3 ± 5.7	25.0 ± 5.3	< 0.001
Obesity (BMI ≥ 30 Kg/m^2^)	322 (13.2 %)	345 (13.5 %)	0.703
Hypertension	325 (13.3 %)	455 (17.7 %)	< 0.001
Hypercholesterolaemia	400 (16.4 %)	560 (21.8 %)	< 0.001
Diabetes mellitus	152 (6.2 %)	146 (5.7 %)	0.422
Renal failure	23 (0.5 %)	39 (0.8 %)	0.065

^a ^The educational level of the participants was measured by the years of schooling (**Group I**: < 9 years, **Group II**: up to high school or technical colleges (10 – 14 years) and **Group III**: university

^b ^**BMI**: Body mass index.

**Table 3:** Prevalence of health conditions and anthropometric indices in relation to hypertension status.

	Normotensive	Hypertensive	P
Hypercholesterolemia	14.8 %	42.7 %	< 0.001
Diabetes mellitus	3.8 %	17.6 %	< 0.001
Renal failure	0.8 %	3.8 %	< 0.001
Obesity	10.8 %	27.3 %	< 0.001
Body mass index (Kg/m^2^)	25.15 ± 5.66	27.99 ± 4.28	< 0.001
Weight (Kg)	73.12 ± 14.29	77.96 ± 13.30	< 0.001

**Table 4:** Distribution of hypertensive study's participants by age group

	Men		Women		P	Total	
Age	N	Hypertensive	N	Hypertensive		N	Hypertensive
18–24	333	5 (1.5 %)	317	2 (0.6 %)	*0.282*	650	7 (1.1 %)
25–34	518	9 (1.7 %)	510	12 (2.4 %)	*0.485*	1028	21 (2.0 %)
35–44	474	25 (5.3 %)	496	31 (6.3 %)	*0.515*	970	56 (5.8 %)
45–54	412	51 (12.4 %)	433	75 (17.3 %)	*0.044*	845	126 (14.9 %)
55–64	358	99 (27.7 %)	405	135 (33.3 %)	*0.090*	763	234 (30.7 %)
65–74	344	136 (39.5 %)	403	200 (49.6 %)	*0.006*	747	336 (45.0 %)

Total	2439	325 (13.3 %)	2564	455 (17.7%)	*< 0.001*	5003	780 (15.6 %)

**Table 5:** Food items consumed (in servings/week), in relation to hypertension status

	Normotensive	Hypertensive	P
Red meat and products	1.63 ± 1.28	1.40 ± 1.13	< 0.001
Pork	1.26 ± 1.15	0.94 ± 0.92	< 0.001
Chicken	1.43 ± 1.10	1.39 ± 0.93	0.298
Fish	1.32 ± 1.09	1.56 ± 1.26	< 0.001
Egg	1.23 ± 1.63	0.87 ± 1.14	< 0.001
Bread and cereals	11.93 ± 8.83	12.79 ± 7.71	0.011
Pasta and rice	2.08 ± 1.47	1.61 ± 1.32	< 0.001
Potatoes	2.23 ± 1.71	1.81 ± 1.55	< 0.001
Vegetables	7.15 ± 3.81	7.31 ± 3.80	0.272
Fruits and juices	7.52 ± 6.26	8.34 ± 6.57	< 0.001
Milk and yogurt (Full Fat)	3.61 ± 4.98	2.53 ± 3.98	< 0.001
Milk and yogurt (Low Fat)	2.38 ± 4.22	3.28 ± 4.70	< 0.001
Yellow cheese	2.22 ± 2.50	1.80 ± 2.36	< 0.001
White cheese	4.99 ± 3.30	4.74 ± 3.18	0.055
Legumes	1.23 ± 0.93	1.16 ± 0.96	0.063
Dessert, ice cream	2.13 ± 2.69	1.41 ± 2.28	< 0.001
